# Efficacy of Empagliflozin Versus Placebo in Patients With Recent Acute Myocardial Infarction: A Systematic Review

**DOI:** 10.7759/cureus.87354

**Published:** 2025-07-05

**Authors:** Carlos A Umaña Mejia, Jose R Flores Valdés, Dominique Alvarado Corletto, Milton A Trujillo Asturias, Alfredo I Rojo Mendoza, Paulina Razo, Jaqueline L Castillo, Diana R Alvarez Flores, Edwin A Garcia-Vasquez, Luis F López Hernández

**Affiliations:** 1 General Medicine, Universidad Autonoma De Guadalajara, Guadalajara, MEX; 2 General Physician, Memorial Hermann Health System, Houston, USA; 3 General Medicine, Universidad Mariano Gálvez De Guatemala, Guatemala City, GTM; 4 General Medicine, Universidad Autonoma De Ciudad Juárez, Ciudad Juarez, MEX; 5 Internal Medicine, Universidad Autonoma De Guadalajara, Guadalajara, MEX; 6 General Medicine, Universidad La Salle, Mexico City, MEX; 7 General Medicine, Universidad San Martin Porres, Chiclayo, PER; 8 General Medicine, Universidad De Guadalajara, Guadalajara, MEX

**Keywords:** acute myocardial infarction, cardiovascular outcomes, empagliflozin, placebo, systematic review

## Abstract

Empagliflozin, a sodium-glucose cotransporter-2 inhibitor (SGLT2), is well-established for managing various cardio-renal conditions, but its effectiveness in patients recovering from acute myocardial infarction (AMI) remains unclear. This systematic review aims to evaluate whether empagliflozin reduces mortality and hospitalization rates compared to placebo in this population. We identified five randomized controlled trials (RCTs) from PubMed and ScienceDirect up to May 2024, which compared empagliflozin to placebo in patients with recent AMI. A total of 7229 participants were included. The primary outcomes were reductions in mortality and hospitalization rates, while secondary outcomes included changes in N-terminal pro-brain natriuretic peptide (NT-proBNP) levels and left ventricular ejection fraction (LVEF).

Empagliflozin did not significantly reduce cardiovascular mortality or hospitalization rates. However, some studies reported a statistically significant reduction in NT-proBNP levels of up to 15% in the empagliflozin group. Additionally, a 1.5% greater increase in LVEF was observed in patients treated with empagliflozin compared to placebo in certain studies. Although empagliflozin has a favorable safety profile and shows positive effects on biomarkers related to heart failure (HF), current evidence does not support its use as an effective intervention for reducing mortality or hospitalizations in AMI patients. Further research is needed to confirm its efficacy in this population.

## Introduction and background

The term acute coronary syndrome (ACS) encompasses conditions involving confirmed or suspected acute myocardial ischemia or infarction, typically resulting from the rupture or erosion of a coronary plaque and thrombus formation, obstructing blood flow [1,2]. Cardiovascular diseases due to atherosclerosis are the leading cause of death in patients with type 2 diabetes. Atherosclerosis is a chronic and progressive condition caused by lipid and fibrous tissue accumulation in major arteries [3].

Each year, ACS affects around one million people in the United States, with an estimated global impact of up to 26 million individuals [4]. Advancements in both medical therapies and interventional strategies have played an important role in reducing mortality rates. Coronary artery disease (CAD) is widely recognized as a major contributor to the development of heart failure (HF), especially following discharge after a myocardial infarction (MI). Post-acute myocardial infarction (AMI) HF is commonly associated with elevated risks of death and hospital readmissions [5]. Traditional cardiovascular risk factors such as high blood pressure, diabetes, smoking, and dyslipidemia are known to significantly increase the likelihood of CAD [6].

Patients with diabetes have an increased risk of cardiovascular complications and often show diminished responses to standard therapies for CAD [7]. Currently, cardiovascular disease affects about 32.2% of individuals with type 2 diabetes mellitus globally, ranking among the most prevalent comorbidities in HF patients [4,8]. There are an estimated 180 million people worldwide who suffer from diabetes. Among emerging treatment options, empagliflozin, a sodium-glucose cotransporter-2 (SGLT2) inhibitor, has gained attention for its potential cardioprotective properties, which are thought to arise from metabolic and anti-inflammatory mechanisms [7,9].

SGLT2 inhibitors have demonstrated a role in lowering HF risk in patients with diabetes who are at high cardiovascular risk. These agents have been shown to reduce hospitalization and mortality, both in individuals with an ejection fraction ≤40% or >40% [10]. Though limited, some studies suggest a favorable impact of empagliflozin on HF outcomes in patients experiencing AMI. Reported benefits include reductions in: cardiovascular-related mortality, non-fatal MI, HF events, and non-fatal strokes [10].

Several mechanisms may explain the positive cardiovascular effects observed with this antidiabetic therapy. These include anti-inflammatory activity, effects on cardiomyocytes, anti-fibrotic and anti-apoptotic processes, improved myocardial metabolism, osmotically induced diuresis, and enhanced sodium excretion [10]. The SGLT2 inhibitor class also appears to support vasodilation by boosting nitric oxide synthesis and availability, contributing to anti-atherosclerotic effects, while additionally inhibiting the Na⁺/K⁺ exchanger [3].

Primarily acting at the level of the renal proximal tubule, SGLT2 inhibitors limit glucose reabsorption, facilitating the excretion of excess glucose in urine. This not only aids in glycemic control but also helps reduce oxidative stress and fibrosis in the heart, potentially reversing atherosclerotic changes [11]. Empagliflozin has also been shown to improve key HF biomarkers and outcomes following AMI, such as lowering natriuretic peptide levels, enhancing left ventricular ejection fraction (LVEF), and reducing cardiac volume overload [12]. Additional advantages associated with this drug class include weight loss, decreased visceral and subcutaneous fat, and lower blood pressure [13]. Evidence also indicates a reduced risk of HF-related hospitalizations, irrespective of a diabetes diagnosis [14].

There is little research available on the use of SGLT2 inhibitors as a cardioprotective alternative or treatment for patients after a MI. This systematic review aims to evaluate the correlation between empagliflozin (SGLT2 inhibitor) and reductions in mortality and hospitalization rates in patients with and without type 2 diabetes after suffering from acute MI compared to placebo.

## Review

Methods 

This systematic review was conducted in accordance with the 2020 Preferred Reporting Items for Systematic Reviews and Meta-Analyses (PRISMA) guidelines, utilizing an evidence-based approach to ensure methodological rigor [15,16]. The review protocol was registered in the International Prospective Register of Systematic Reviews (PROSPERO) database under the registration number CRD42024578524.

*Search Methods* 

To ensure the inclusion of only high-quality evidence, stringent selection criteria were implemented. Exclusion criteria were strictly enforced to preserve the relevance and integrity of the studies under review. Studies were omitted if they did not specifically examine the relationship between empagliflozin and its impact on mortality and hospitalization rates in individuals with or without type 2 diabetes following AMI, compared to placebo. Furthermore, articles that were not accessible in full text or unobtainable through interlibrary loan services were excluded. A comprehensive literature search was conducted across several databases using a combination of Medical Subject Headings (MeSH) and free-text keywords tailored to the research objective. The selection of studies followed a structured process, illustrated by a PRISMA flow diagram, to ensure transparency and consistency. This methodical approach allowed for the development of a uniform dataset, supporting a more accurate synthesis of findings.

A systematic review of the literature was carried out to evaluate and compare the effectiveness of empagliflozin versus placebo in patients who had recently experienced an AMI. The primary outcomes assessed included hospitalization rates, the combined incidence of hospitalization for heart failure (HHF) and cardiovascular death, and all-cause mortality. The search spanned publications from 2019 to May 2024, drawing from databases such as PubMed and ScienceDirect. A strategic combination of keywords was employed, including terms like “adult,” “acute myocardial infarction,” “SGLT2 inhibitors,” and “empagliflozin”.

Selection criteria

Type of Participants 

This review established criteria that include (a) participants were 18 years of age or older and diagnosed with ACS; (b) the experimental group was administered empagliflozin, whereas the control group received a placebo; and (c) studies that included patients other than those with ACS were excluded. We excluded studies involving pediatric populations, patients under 18, and pregnant patients. 

*Types of Intervention* 

We selected studies that met the inclusion criteria. The interventions may include the administration of empagliflozin versus placebo in patients with recent AMI. 

Types of Study 

Data preparation and analysis were conducted in alignment with the PRISMA guidelines. A thorough search of PubMed and ScienceDirect was carried out, focusing on literature published between 2019 and May 2024. This review included randomized controlled trials (RCTs), cohort studies, and case-control studies that evaluated the effectiveness of empagliflozin compared to placebo in patients who had recently experienced an AMI. Studies such as case reports, case series, cross-sectional studies, dissertations, book chapters, protocol papers, reviews, news articles, conference abstracts, letters to the editor, editorials, and commentaries were excluded. Additionally, any RCTs, cohort, or case-control studies published more than five years prior to the search window were omitted. 

*Types of Outcomes * 

In our systematic review, our primary endpoint of interest was the hospitalization rate and the combination of HHF with cardiovascular mortality. Secondary outcomes included overall mortality, rehospitalization rate, rate of patients with HF, glomerular filtration rate of patients treated with empagliflozin vs placebo, rate of patients treated with fibrinolytics and percutaneous coronary intervention, recurrent ACS, changes in post-treatment natriuretic peptide value, and changes in echocardiography. 

Selection of Studies, Data Extraction, and Screening

Two reviewers (DAC and CAUM) utilized Rayyan for the initial screening of titles and abstracts [17]. To ensure consistency, a third independent reviewer (PMRB) assessed the relevance of each study based on the predefined inclusion and exclusion criteria. This was followed by a full-text evaluation, during which two additional reviewers (EGV and CAUM) independently reviewed the selected articles using the same eligibility criteria. Any discrepancies that arose during this phase were resolved by consensus, with input from a third reviewer (DRAF) when needed. All studies identified during the search process were evaluated for relevance, and those deemed potentially eligible underwent a detailed assessment to determine their inclusion or exclusion based on the established criteria. 

*Data Evaluation: Assessment of Risk of Bias * 

The evaluation process adhered to the guidelines set in the Cochrane Handbook for Systematic Reviews of Interventions [18]. Risk of bias in RCTs was assessed using the Cochrane Risk of Bias Tool for RCTs [19]. Two independent reviewers (MATA, PMRB) conducted the bias assessments, applying the tool’s criteria to each study. Any disagreements were addressed through discussion, with resolution provided by a third, blinded reviewer (DRAF). As outlined in the Cochrane Handbook, the included studies exhibited no bias or some concerns with methodological bias. All randomized trials reported their randomization procedures, and several explicitly stated that intervention providers and participants were blinded to group assignments.

Results

We conducted a systematic literature review to assess and compare the efficacy of empagliflozin versus placebo in patients with recent AMI. The study focused on hospitalization rate, the combination of HHF with cardiovascular mortality, and overall mortality. Our search, covering the period from 2019 to May 2024, encompassed databases such as PubMed and ScienceDirect.

A systematic review relies fundamentally on the thorough identification and selection of relevant studies from a broad literature base. Our process began with an extensive database search, which yielded a total of 947 records. After verifying for duplicates, none were identified. Following a structured screening of titles and abstracts, 910 studies were excluded due to irrelevance or failure to meet inclusion criteria. The remaining 37 articles were subjected to full-text review, ultimately resulting in the inclusion of five high-quality studies that met all predefined eligibility standards for final synthesis.

**Figure 1:**
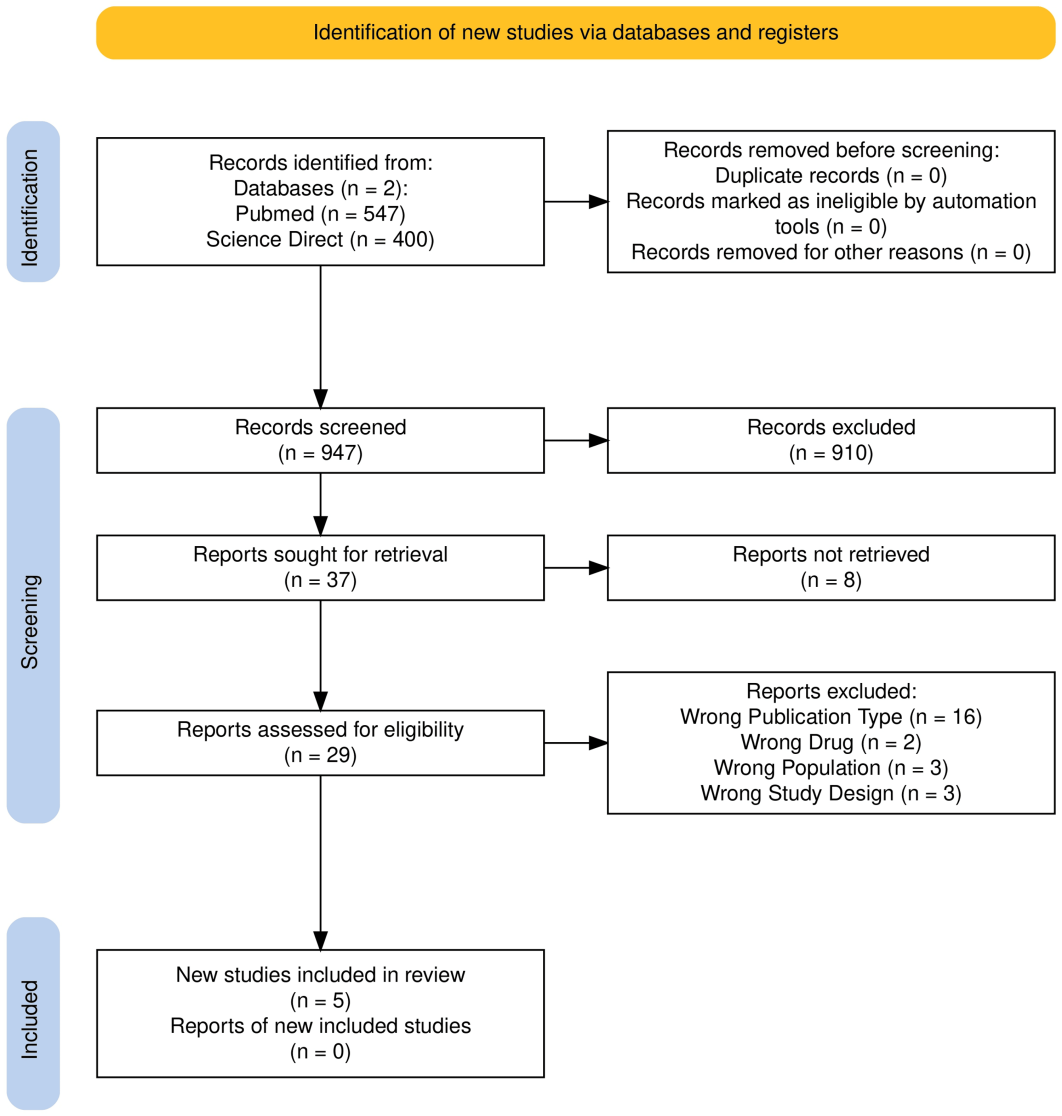
Flow diagram of our bibliographic search

Figure 1 provides a clear visual representation of the study selection process, structured according to the PRISMA flow diagram format [15]. This illustration offers a transparent overview of each screening stage, culminating in the final inclusion of studies. 

This flowchart depicts the sequential stages of literature screening conducted in accordance with the PRISMA framework, demonstrating the process of identifying and selecting studies relevant to this systematic review [15]. A total of 947 records were retrieved from PubMed and ScienceDirect. After confirming the absence of duplicates, 910 articles were excluded during the initial screening phase. This left 37 articles for full-text review; however, eight could not be accessed, leaving 29 studies for eligibility assessment. Of these, 24 were excluded for various reasons: unsuitable publication type (n=7), irrelevant population (n=3), incompatible study design (n=1), and non-relevant drug interventions (n=1). Ultimately, five studies fulfilled the inclusion criteria and were included in the final analysis. The flowchart below illustrates this selection process, reflecting the thorough and systematic approach employed to ensure the relevance and quality of evidence.

**Figure 2:**
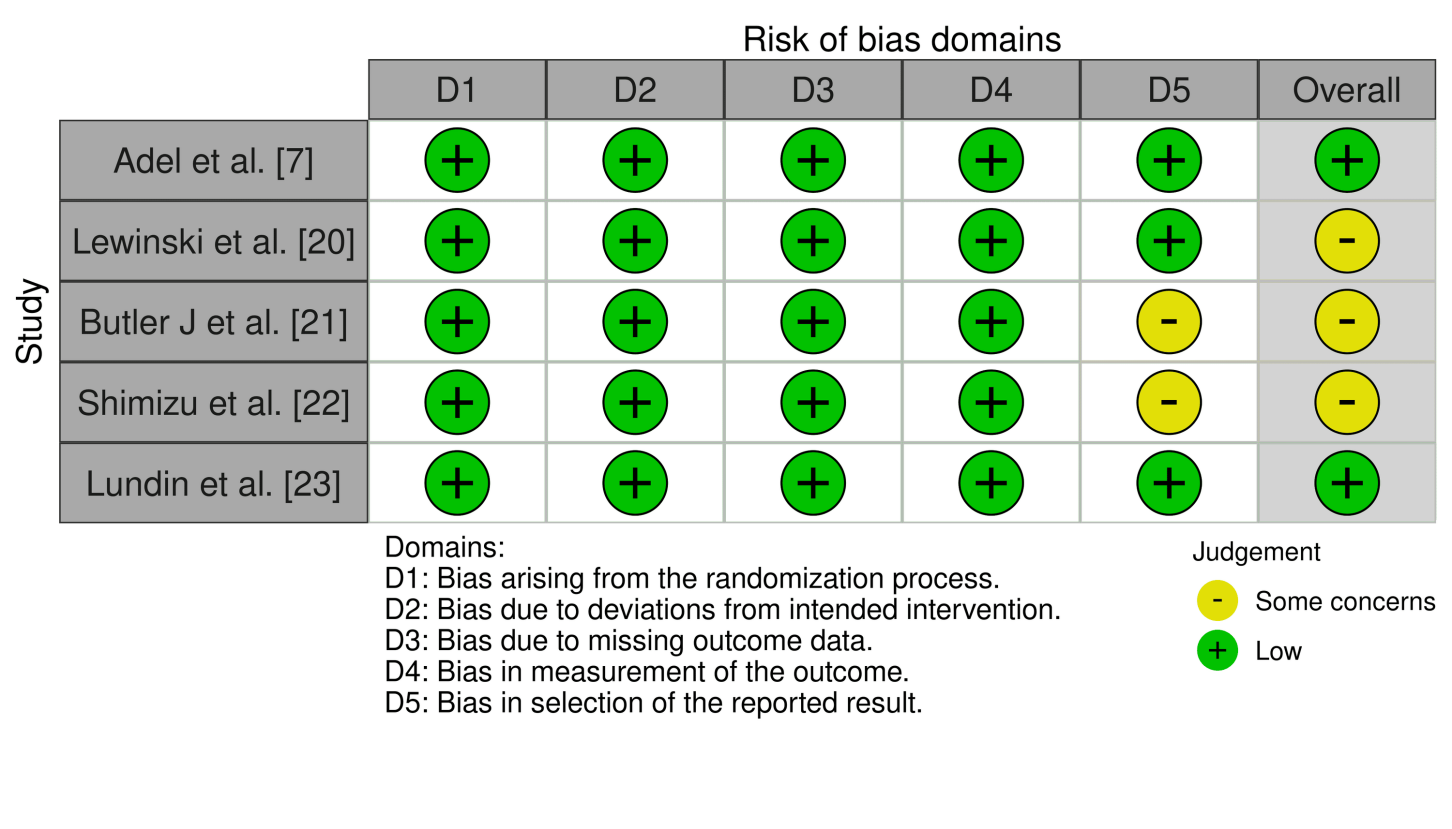
Risk of bias Each article was assessed for risk of bias. Of the five articles evaluated, three exhibited some concerns regarding overall bias, while two showed a low risk of bias [7,19-23].

**Figure 3:**
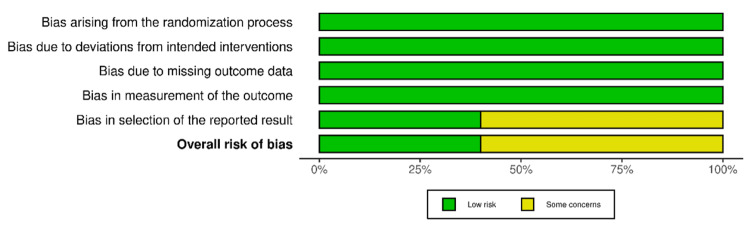
Summary plot for RCTs This diagram shows part of the risk of bias assessment, with the overall results represented in two colors: green (low risk) and yellow (some concerns) [19]. RCT: randomized controlled trial

Risk of bias was evaluated using the Cochrane Risk of Bias 2.0 Tool for RCTs [19]. As summarized in Figure 2 and Figure 3, two studies were judged to have a low risk of bias, while three studies raised some concerns, primarily related to incomplete reporting of outcome data.

Of the five studies examined, all were RCTs, and the quality of the studies was generally high according to the ROB 2.0 ​(Risk of Bias Tools - Robvis (Visualization Tool) [19,24]​. In the five studies evaluated, there was a total population of 7229 patients. Four of the five studies had an intake of 10 mg of empagliflozin and only one had an intake of 25 mg, both groups with a similar sample size with a minimum variation of two to four patients, these groups were made up of similar age range from 55 to 68 years old who had a history of a recent AMI, as well as a history of cardiovascular diseases and risk factors such as arterial hypertension, DM2 and positive smoking among others. However, all the studies included in this research showed that empagliflozin was not significantly different from placebo. The main objective of all the studies was to evaluate the efficacy of empagliflozin intake in reducing the mortality rate and decreasing rehospitalizations after having recently suffered an AMI. When measuring the mortality rate across all studies, it was found that empagliflozin was not significantly better than placebo. Only two of the five studies showed an increase in the mortality rate with empagliflozin. Lewinsky reported three deaths in the empagliflozin group, while no deaths were reported in the placebo group [20].

Based on the number of hospitalizations, it was also shown that a significant variation was not obtained; however, in two of the five articles evaluated, a decrease in hospitalizations with empagliflozin was shown. Lewinsky reported 31 hospitalizations in the empagliflozin group, while 32 hospitalizations occurred in the placebo group. In Adel's study, two hospitalizations were reported in the empagliflozin group, compared to four in the placebo group [7,20].

In terms of secondary outcomes, we analyzed changes in N-terminal pro-brain natriuretic peptide (NT-proBNP) values post-treatment. Lewinski reported a 15% reduction in NT-proBNP values in the empagliflozin group compared to the placebo group [20]. Shimizu also observed a significant decrease in NT-proBNP levels at 24 weeks in the empagliflozin group [22]. In contrast, Lundin et al., who utilized a greater dose of 25 mg, found no significant influence of empagliflozin on NT-proBNP values, suggesting that the response to empagliflozin may vary across cohorts and doses [23]. We also evaluated the LVEF. Lewinski reported a 1.5% increase in LVEF in the empagliflozin group compared to the placebo group, suggesting a positive impact on left ventricular function [20]. However, Adel found a 5% increase in LVEF in both the empagliflozin and placebo groups, but without statistical significance, indicating that LVEF benefits may not be consistent across all studies [7].

In conclusion, with all data summarized in Table 1, the results indicate that there was no significant response in the use of empagliflozin compared to the use of placebo regarding our primary outcome, but significant responses were found for secondary outcomes. 

**Table 1:** The general outcomes are summarized in the table N/A: not applicable; RCT: randomized control trial; MI: myocardial infarction; DM2: diabetes mellitus type 2; AE: adverse events; F/M: female/male; CABG: coronary artery bypass graft; ECG: electrocardiography; eGFR: estimated glomerular filtration rate; HRT: heart rate turbulence; HRV: heart rate variability; LP: late potentials; TWA: T-wave alternans; NT-Pro BNP: N-terminal pro b-type natriuretic peptide; BW: body weight; BP: blood pressure; TIA: transitory ischemic attack; CKD: chronic kidney disease LVEDV: left ventricular end diastolic volume; LVEF: left ventricular ejection fraction; AST: aspartate aminotransferase; ALT: Alanine aminotransferase; LDL: Low density lipoprotein; HDL: High density lipoprotein; CAD: coronary artery disease; HF: heart Failure

Author, year	Lewinski et al., 2022 ​[20]	Butler J et al., 2024 ​[21]	Adel et al., 2022 ​[7]	Shimizu et al., 2020 ​[22]	Lundin et al., 2022 ​[23]
Study type	RCT	RCT	RCT	RCT	RCT
Total participants in the study	476	6522	93	96	42
# in intervention group (empagliflozin)	237	3260	45	46	20
# in placebo group	239	3262	48	50	22
Empagliflozin dose	10 mg	10 mg	10 mg	10 mg	25 mg
Mean age of intervention	57 (52-64)	63.6+11.0	55 (45.5-64)	63.9+10.4	67+8
Mean age of placebo	57 (52-65)	63.7+10.8	57 (50-66.75)	64.6+11.6	68+8
# of F/M in intervention group	Males: 195; females: 42	Females: 812; males: 2448	Males: 27; females: 18	Males: 38; females: 8	Males: 16; females: 4
# of F/M in placebo group	Males: 197; females: 42	Females: 813; males: 2449	Males: 29; females: 19	Males: 39; females: 11	Males: 18; females: 4
F/M race or ethnic group # (%) intervention group	N/A	Caucasian: 2730 (83.7%); African American: 44 (1.3%); and Asian: 421 (12.9%)	N/A	Asian: 46 (47.9%)	N/A
F/M race or ethnic group # (%) placebo group	N/A	Caucasian: 2721 (83.4%); African American: 48 (1.5%); and Asian: 413 (12.7%)	N/A	Asian 50 (52%)	N/A
Cardiovascular disease history and risk factors (#) intervention group	Hypertension: 92; DM2: 30; obesity: 68; smoking: 171; CAD: 28; history of stroke: 5; history of CABG: 1; history of MI: 14; and coronary angiograph vessel status: 1 vessel (105), 2 vessels (82), and 3 vessels (50)	Previous MI: 388 (11.9%); atrial fibrillation: 358 (11%); DM2: 1035 (31.7); hypertension: 2262 (69.4%); and peripheral artery disease: 172 (5.3%)	Smoking: 9; CDK: 4; hypertension: 26; and history of stroke: 1	Cerebrocardiovascular disease: 7 (15.2%); hypertension: 38 (82.6%); and dyslipidemia: 34 (73.9%)	Peripheral artery disease: 1; stroke/TIA: 2; and HF: 1
Cardiovascular disease history and risk factors (#) placebo group	Hypertension: 107; DM2: 63; obesity: 107; smoking: 170; CAD: 25; history of stroke: 1; history of CABG: 1; history of MI: 9; coronary angiography vessel status: 1 vessel (123), 2 vessels (80), and 3 vessels (36)	Previous MI: 459 (14.1%); atrial fibrillation 361 (11.1%); DM2: 1046 (32.1%); hypertension: 2276 (69.8%); and peripheral artery disease: 180 (5.5)	Smoking: 8; CDK: 3; hypertension: 32; and history of stroke: 2	Cerebrocardiovascular disease: 11 (22.0%); hypertension: 39 (78,0%); and dyslipidemia: 36 (72,0%)	Peripheral artery disease: 0; stroke/TIA: 0; HF: 0
Biomarkers	Creatine kinase, troponin T, total cholesterol, LDL, HDL NT-proBNP, AST, ALT, and hemoglobin A1c	N/A	Hemoglobin A1c, fasting blood sugar, and LDL	Holter ECG with markers: as HRV, HRT, LP, and TWA, glycemic and lipid profiles, uric acid, eGFR, BW, BP, creatinine, hematocrit, NT-pro BNP, AST, and ALT	Troponin, creatine, LDL, triglycerides, HbA1c, NT-proBNP, fasting plasma glucose, and 2-hour post-load glucose
Follow-up time	26 weeks	17.9 months	6 months	6 months	10 months
# of death in intervention group	3	169 (5.2%)	1	0 (0%)	N/A
# of death is placebo group	0	298 (9.1%)	2	0 (0%)	N/A
AEs in intervention group	Hospitalization due to HF: (3), hospitalization due to cardiovascular event (2), hepatic injury (1), renal injury (0), urinary tract infection (11), genital fungal infection (7), deaths from cardiovascular cause (2), and non cardiovascular death (1)	Ketoacidosis: 2 (0.1%; AE leading to lower limb amputation: 9 (0.3%); hepatic Injury: 8 (0.2%); acute renal failure: 43 (1.3%); acute kidney injury: 27 (0.8%); and hypoglycemia: 4 (0.1%)	Deaths from cardiovascular cause: 1	0 (0%)	N/A
AEs in placebo group	Hospitalization due to heart failure (4), hospitalization due to cardiovascular event (5), hepatic injury (1), urinary tract infection (7), genital fungal infection (2), deaths from cardiovascular cause (0), and non-cardiovascular death (0)	Ketoacidosis: 1 (0.1%); AE leading to lower limb amputation: 5 (0.2%); hepatic Injury: 2 (0.1%), acute renal failure: 59 (1.8%); acute kidney injury: 43 (1.3%); and hypoglycemia: 5 (0.2%)	Death from cardiovascular cause: 2	Dizziness: 1 (2.0%); HF 1 (2.0%); abdominal pain: 1 (2.0%); hepatic impairment: 1 (2.0%); and rash 1 (2.0%)	N/A
# of hospitalization in intervention group	31 (35%)	317	Hospitalized due to unstable angina: 2	N/A	N/A
# of hospitalization in placebo group	32 (34%)	298	Hospitalized due to unstable angina: 4	1	N/A
Key points	A 1.5% greater increase in LVEF was found in the empagliflozin group when compared to the placebo group (95% CI, 0.2 to 2.9%; P=0.029). As well as a 15% lower NT-proBNP value in the empagliflozin group compared to the placebo group (95% CI, -4.4% to –23.6%, P=0.026)	No significant variation was seen regarding mortality and hospitalizations in the empagliflozin group compared to the placebo group	A 5% increase in LVEF was seen in both the empagliflozin group and the placebo group but with no statistical significance	The initial mean NT-proBNP value in the empagliflozin group was 1028.7 ± 1105.6, which decreased to 370.3±530.9 at the 24-week follow-up (p<0.001). In the placebo group, the baseline NT-proBNP value was 1270.6±1521, which reduced to 672.7±1151 (p<0.001), demonstrating a greater reduction in the empagliflozin group	Empagliflozin did not influence LVEDV and NT-proBNP values in a significant manner

Discussion

Empagliflozin, a SGLT2 inhibitor, has been shown to improve cardiovascular outcomes in patients with type 2 diabetes and HF, independent of its glucose-lowering effects. Its mechanisms include natriuresis, reduction in preload and afterload, improved myocardial energy metabolism, and attenuation of cardiac fibrosis, all of which may benefit patients recovering from AMI [25]. These potential mechanisms have led to growing interest in exploring its use in post-AMI management, particularly for preventing the development of HF and other adverse cardiovascular events.

The studies analyzed in this systematic review have provided a comprehensive view of the efficacy of empagliflozin compared to placebo in patients with recent AMI. The main objective of our review was to assess whether empagliflozin effectively reduces mortality rates and decreases hospitalizations following an AMI. Five studies were included, with a combined population of 7229 patients. In four of the five studies, patients received 10 mg of empagliflozin, while one study used a dose of 25 mg. Notably, no studies directly compare the benefits of 10 mg versus 25 mg doses of empagliflozin, highlighting a potential area for future research. The age of the study population ranged from 45 to 75 years, limiting the evaluation of treatment effects in younger or older patients outside this age range. 

When analyzing mortality rates across all studies, no significant improvement was observed compared to placebo. Butler et al. reported 169 deaths in the empagliflozin group versus 178 in the placebo group. Cardiovascular-related deaths were 132 in the empagliflozin group and 131 in the placebo group [21]. Additionally, Adel et al. reported one death in the empagliflozin group and two in the placebo group, while Lewinski et al. reported three deaths in the empagliflozin group and none in the placebo group [7,20]. 

Regarding hospitalizations, the studies did not demonstrate a significant overall variation; however, three of the articles indicated a reduction in hospitalizations with empagliflozin. Butler et al. reported 118 initial hospitalizations for HF in the empagliflozin group compared to 153 in the placebo group [21]. Lewinski et al. observed 31 hospitalizations in the empagliflozin group, including three for HF and two for cardiovascular events, compared to 32 hospitalizations in the placebo group, with four for HF and five for cardiovascular events [20]. Adel et al. reported two hospitalizations in the empagliflozin group and four in the placebo group due to unstable angina [7]. 

The overall analysis of the studies included in this review demonstrates that among patients at higher risk of HF after an AMI, treatment with empagliflozin did not result in a significantly lower risk of first hospitalization for HF or death from any cause compared to placebo. Butler J et al. also found no significant differences in mortality and hospitalizations between the empagliflozin and placebo groups [21]. This finding is critical as it suggests that while empagliflozin may have beneficial effects on certain biomarkers and cardiac functions, these do not necessarily translate into a reduction in adverse clinical events such as mortality or hospitalizations. 

It is important to consider the limitations of the included studies, such as sample size and follow-up duration. Some studies have relatively small sample sizes and short follow-up periods, which may limit the ability to detect significant differences in important clinical outcomes. Additionally, variability in the studied populations, including differences in baseline patient characteristics, such as the presence of comorbidities and concurrent treatments, can influence the observed outcomes and complicate direct comparisons between studies. Clinicians should interpret the current evidence with caution, especially when considering empagliflozin in patients without diabetes or established HF. While the improvements in biomarkers such as NT-proBNP are encouraging, they should not yet be the sole basis for treatment decisions until more conclusive outcome data are available.

Despite these limitations, the results suggest that empagliflozin may offer additional benefits in managing patients with AMI, particularly in terms of improving ventricular function and reducing markers of cardiac stress. Future studies with larger sample sizes and longer follow-up periods are needed to confirm these findings and assess their impact on long-term mortality. 

## Conclusions

Although empagliflozin has a favorable safety profile and has shown positive effects on certain biomarkers related to HF, current evidence does not support its use as an effective intervention to reduce mortality or hospitalizations in patients with recent AMI. To fully determine the potential of empagliflozin in this patient population, additional studies with larger sample sizes and longer follow-up periods are needed. These studies should focus on evaluating its impact on long-term mortality and morbidity. In conclusion, while empagliflozin shows promise as an adjunctive therapy in the management of post-AMI, more evidence is required to establish its definitive role in clinical practice. 
